# Prostate cancer in firefighting and police work: a systematic review and meta-analysis of epidemiologic studies

**DOI:** 10.1186/s12940-017-0336-z

**Published:** 2017-11-17

**Authors:** Jeavana Sritharan, Manisha Pahwa, Paul A. Demers, Shelley A. Harris, Donald C. Cole, Marie-Elise Parent

**Affiliations:** 1 Occupational Cancer Research Centre, Cancer Care Ontario, 525 University Avenue, Toronto, ON M5G 2L3 Canada; 2 Institute of Medical Science, University of Toronto, 525 University Avenue, Toronto, ON M5G 2L3 Canada; 3 CAREX Canada, Simon Fraser University, Burnaby, Canada; 4 Dalla Lana School of Public Health, University of Toronto, 525 University Avenue, Toronto, ON M5G 2L3 Canada; 5 Population Health and Prevention, Cancer Care Ontario, 525 University Avenue, Toronto, ON M5G 2L3 Canada; 6 INRS-Institut Armand-Frappier, University of Quebec, 531 Boulevard des Prairies, Laval, Quebec, H7V 1B7 Canada

**Keywords:** Firefighters, Police, Occupation, Prostate cancer risk, Incidence, Mortality, Meta-analysis, Systematic review, Epidemiology

## Abstract

**Objectives:**

We conducted a systematic review and meta-analysis to evaluate potential associations between firefighting and police occupations, and prostate cancer incidence and mortality.

**Methods:**

Original epidemiological studies published from 1980 to 2017 were identified through PubMed and Web of Science. Studies were included if they contained specific job titles for ever/never firefighting and police work and associated prostate cancer risk estimates with 95% confidence intervals (CI). Study quality was assessed using a 20-point checklist. Prostate cancer meta-risk estimates (mRE) and corresponding 95% CIs were calculated for firefighting and police work separately and by various study characteristics using random effects models. Between-study heterogeneity was evaluated using the I^2^ score. Publication bias was assessed using Begg’s and Egger’s tests.

**Results:**

A total of 26 firefighter and 12 police studies were included in the meta-analysis, with quality assessment scores ranging from 7 to 19 points. For firefighter studies, the prostate cancer incidence mRE was 1.17 (95% CI = 1.08–1.28, I^2^ = 72%) and the mortality mRE was 1.12 (95% CI = 0.92–1.36, I^2^ = 50%). The mRE for police incidence studies was 1.14 (95% CI = 1.02–1.28; I^2^ = 33%); for mortality studies, the mRE was 1.08 (95% CI = 0.80–1.45; I^2^ = 0%). By study design, mREs for both firefighter and police studies were similar to estimates of incidence and mortality.

**Conclusion:**

Small excess risks of prostate cancer were observed from firefighter studies with moderate to substantial heterogeneity and a relatively small number of police studies, respectively. There is a need for further studies to examine police occupations and to assess unique and shared exposures in firefighting and police work.

**Electronic supplementary material:**

The online version of this article (10.1186/s12940-017-0336-z) contains supplementary material, which is available to authorized users.

## Background

Prostate cancer is one of the most commonly diagnosed cancers in men worldwide but its etiology remains poorly understood [1–5]. The only established risk factors for prostate cancer are older age, positive family history of prostate cancer, and African-American ethnicity [1, 2, 4, 5]. There is some evidence linking prostate cancer to differences in socioeconomic status, increased height, increased obesity, reduced physical activity, and active smoking and alcohol use [3, 5–10]. There is growing evidence that occupation may be a risk factor, and previous studies have shown increased risks associated with employment in agriculture/farming, management and administration, rubber production, metal work, and transportation [11–13]. Some studies have also suggested associations between prostate cancer risk and employment in protective services occupations [11, 12, 15–17].

Protective services occupations include firefighting, police, military, and other groups (eg. security guards). Previous epidemiological studies have demonstrated consistent associations between firefighting and different types of cancer, with some evidence for prostate cancer [14]. In 2007, the International Agency for Research on Cancer (IARC) classified firefighting as “possibly” carcinogenic to humans (IARC Group 2B) [16]. IARC’s evaluation was based on evidence from 42 epidemiological studies, including two previous meta-analyses on firefighting and cancer [14, 18]. Based on studies published at the time, IARC evaluated multiple cancer sites and identified statistically significant increased risks of prostate cancer, testicular cancer, and non-Hodgkin lymphoma [16]. Since the IARC evaluation, 11 new studies have been published that included assessments of prostate cancer risk in firefighters. Relatively less is known about prostate cancer risk in police occupations, as this group is often understudied and findings have been inconsistent [11, 12, 15, 19, 20].

Only one meta-analysis, published over a decade ago, focused on firefighting and cancer risks that included prostate cancer [14]. This study found a significant association with prostate cancer incidence (summary risk estimate: 1.28, 95% CI: 1.15–1.43) based on evidence from 6 cohort studies [14]. Recently, a narrative review examined cancer risk in police work. Eight studies reported on prostate cancer risk in police work, with mixed findings [15]. The objective of the present systematic review and meta-analysis was to evaluate the quality of the epidemiological evidence on firefighting and police employment in association with prostate cancer incidence and mortality, and to conduct a quantitative synthesis. Based on the availability of epidemiologic literature, this meta-analysis focused on firefighting and police work, and not protective services as a whole.

## Material and methods

### Search strategy

A search was conducted on PubMed and Web of Science to identify epidemiological studies published between January 1980 and December 2017 in English or French about employment in firefighting and police occupations, and risk of prostate cancer. Various combinations of MeSH terms were used to search for studies that included firefighter and police occupations (firefighting OR firefighter OR fire fighter OR fire OR police OR police officer OR policeman OR policemen) and that reported on associations with prostate cancer risk (prostate OR prostate neoplasm OR neoplasm OR cancer). Cited references in individual papers and review papers that resulted from the search were used to identify any additional studies.

### Inclusion criteria

To be included in the meta-analysis, articles must have reported results for original case–control or cohort studies that contained specific job titles related to ever/never firefighting and police work and that examined associated prostate cancer incidence and/or mortality using any type of relative risk estimator (hazard ratio (HR), odds ratio (OR), relative risk (RR), standardized mortality ratio (SMR), or standardized incidence ratio (SIR)) with corresponding 95% confidence intervals. Reviews, meta-analyses, editorials, and experimental studies were excluded. For any articles with overlapping study populations, only the most recently published study with prostate cancer incidence and/or mortality results was included. Furthermore, studies were excluded if reported risk estimates were only based on internal comparisons between different occupational groups rather than based on comparisons to the general population. Titles and abstracts were initially screened for eligibility, and for those eligible, full-text articles were reviewed.

### Data extraction

Information on author(s), date of publication, title, country of study, study design, number of cases/deaths and controls/non-cases, data collection method, effect sizes and 95% CIs for prostate cancer, and covariates was extracted from and tabulated for each study included in the meta-analysis. Effect sizes and 95% Cls recorded from included studies were for ever vs. never firefighter or police employment in models that were adjusted for the maximum number of potentially confounding variables.

### Quality assessment

The quality of each study included in the meta-analysis was independently assessed by two authors (JS and MP) using a modified quality assessment checklist by Downs and Black [21]. Checklist items that were irrelevant to observational studies were omitted, resulting in a maximum of 20 achievable points for reporting (9 points), external validity (2 points), internal validity (bias and confounding) (8 points), and power (1 point) [21]. Any disagreement of ratings was discussed and a consensus was arrived at mutually or by consulting a third author, if earlier consensus could not be reached.

### Statistical analysis

Reported ORs, HRs, RRs, SIRs, and SMRs were considered as RRs in meta-analyses and used in forest plots. A random effects model was used to calculate meta-risk estimates (mREs) in all meta-analyses due to potential variance in effect sizes between the included studies. mREs were calculated separately for firefighting and police occupations and prostate cancer risk. mREs were calculated for subgroups based on the following characteristics: incidence versus mortality, study design (i.e. cohort versus case–control, and administrative linkage-based studies, defined as large studies that used multiple linked administrative databases, e.g. census data and tumour registries.

For each mRE, heterogeneity was evaluated using the I^2^ statistic. The I^2^ statistic is a percentage that describes the variation between studies that is not due to chance [22]. Two-sided *p*-values for the I2 statistic were reported. Ninety-five percent confidence intervals for the I2 statistic were calculated to address small numbers of included studies (*N* < 5) in some subgroup meta-analyses. In addition, the Galbraith plot was used to visualize if individual studies fell within or outside of the 95% confidence region. Studies outside of the 95% confidence region can contribute to high heterogeneity. These studies were removed in sensitivity analyses to evaluate the impact of decreased heterogeneity on mREs [23].

Begg’s test and Egger’s test were used to assess publication bias. Begg’s test uses the correlation between ranks of effect sizes and variances, whereas Egger’s test uses a funnel plot to plot the effect estimates against sample size [24, 25]. All statistical analyses were performed using STATA version 14.2 (StataCorp LLC, College Station, USA).

## Results

**Fig. 1:**
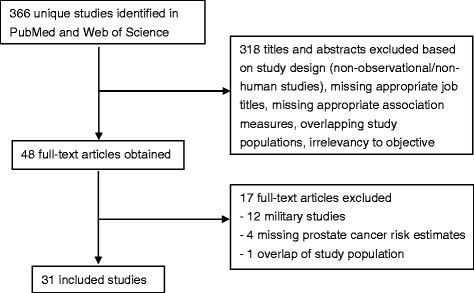
Descriptive flow chart of study selection in this meta-analysis

The literature search resulted in 366 unique studies published in English or French. Based on the screening of titles and abstracts, 318 (87%) were excluded due to non-observational/non-human studies, missing job titles, missing effect estimates for prostate cancer, duplicate studies, or irrelevancy to the objective of this meta-analysis. Of the remaining 48 studies that were obtained in full text, 17 were excluded because they did not include reports of relative risks for prostate cancer with 95% CIs, had overlapping study populations, or were studies of military workers. As a result, 31 unique studies were included (Fig. 1).

Of these, 24 were cohort and seven were case–control studies. Nineteen studies only included investigations of firefighters (Table 1) and five focused on police workers (Table 2); seven contained investigations of both firefighters and police workers (Table 3). In all studies that included firefighters (*N* = 26), there were 5712 incident cases of prostate cancer and 428 deaths from prostate cancer. In all studies that included police workers (*N* = 12), there were 1510 incident cases and 49 deaths. The characteristics of each included study are summarized in Tables 1 (firefighters), 2 (police workers), and 3 (both). Covariates included in the risk estimates selected from each of the seven case–control studies are shown in Additional file 1: Table S1.

**Table 1:** Characteristics of included studies on firefighting and prostate cancer risk (*N* = 19)

Author/Year	Location of Study	Study Design	Incidence or Mortality	Follow-up period	Number of Cases/Deaths	Cohort Size/Total Number of Cases^a^	Prostate Cancer Risk Estimates for Ever versus Never Employment^b^
Glass et al. 2016 [63]	Australia	Cohort	Incidence	1980–2011	478	30, 057	SIR 1.31, 95% CI 1.19–1.43
Brice et al. 2015 [64]	France	Cohort	Mortality	1979–2008	17	10, 829	SMR 0.54, 95% CI 0.31–0.86
Daniels et al. 2014 [32]	USA	Cohort	Incidence; Mortality	1950–2009	1261 cases 282 deaths	29, 993 29, 993	SIR 1.03, 95% CI 0.98–1.09; SMR 1.09, 95% CI 0.96–1.22
Pukkala et al. 2014 [17]	Denmark, Finland, Iceland, Norway and Sweden	Cohort (linkage)	Incidence	1961–2005	660	16, 422	SIR 1.13, 95% CI 1.03–1.22
Ahn et al. 2012 [65]	Korea	Cohort	Incidence	1996–2007	9	33, 416	SIR 1.32, 95% CI 0.60–2.51
Ma et al. 2006 [27]	USA	Cohort	Incidence	1981–1999	209	34, 796	SIR 1.10, 95% CI 0.95–1.42
Ma et al. 2005 [26]	USA	Cohort	Mortality	1972–1999	21	34, 796	SMR 1.08, 95% CI 0.67–1.65
Baris et al. 2001 [66]	USA, USA	Cohort	Mortality	1925–1986	31	7, 789	SMR 0.96, 95% CI 0.68–1.37
Bates et al. 2001 [67]	New Zealand	Cohort	Incidence	1977–1995	11	4, 221	SIR 1.08, 95% CI 0.50–1.90
Tornling et al. 1994 [31]	Sweden	Cohort	Incidence; Mortality	1951–1986	28 cases 14 deaths	1, 116 1, 091	SMR 114, 95% CI 76–165; SMR 121, 95% CI 66–202
Aronson et al. 1994 [68]	Canada	Cohort	Mortality	1950–1989	16	5, 373	SMR 132, 95% CI 76–215
Giles et al. 1993 [69]	Australia	Cohort	Incidence	1980–1989	5	2, 865	SIR 2.09, 95% CI 0.67–4.88
Guidotti 1993 [70]	Canada	Cohort	Mortality	1927–1987	8	3, 328	SMR 146.1, 95% CI 63.1–287.9
Beaumont et al. 1991 [33]	USA	Cohort	Incidence	1940–1982	8	3, 066	RR 0.38, 95% CI 0.16–0.75
Grimes et al. 1991 [71]	USA	Cohort	Mortality	1969–1988	4	205	PRR 2.6, 95% CI 1.4–5.0
Vena & Friedler 1987 [72]	USA	Cohort	Mortality	1950–1979	5	470	SMR 0.71, 95% CI 0.23–1.65
Tsai et al. 2015 [73]	USA	Case–control (linkage)	Incidence	1988–2007	1397	3, 996	OR 1.45, 95% CI 1.25–1.69
Kang et al., 2008 [74]	USA	Case–control (linkage)	Incidence	1986–2003	577	285, 964	SMOR 1.05, 95% CI 0.88–1.24
Krstev et al. 1998 [75]	USA	Case–control	Incidence	1986–1989	12	981	OR 3.34, 95% CI 1.13–9.91

^a^cohort size represents the total sample size in only cohort studies, and the total number of cases is only applicable to case–control studies

^b^HR – hazard ratio, SIR – standardized incidence ratio, SMR – standardized mortality/morbidity ratio, RR – relative risk, PRR – proportionate risk ratio, OR – odds ratio, NR – not reported

**Table 2:** Characteristics of included studies on police work and prostate cancer risk (*N* = 5)

Author/Year	Location of Study	Study Design	Incidence or Mortality	Follow-up Period	Number of Cases/Deaths	Cohort Size/Total Number of Cases^a^	Prostate Cancer Risk Estimates for Ever versus Never Employment^b^
Vena et al. 2014 [19]	USA	Cohort	Mortality	1980–2005	31	3, 424	SMR 1.18, 95% CI 0.80–1.67
Gu et al. 2011 [76]	USA	Cohort	Incidence	1976–2006	104	2, 234	SIR 0.88, 95% CI 0.72–1.07
Finkelstein 1998 [20]	Canada	Cohort	Incidence	1964–1995	85	22, 197	SIR 1.16, 95% CI 0.93–1.43
Forastiere et al. 1994 [77]	Italy	Cohort	Mortality	1973–1991	7	3, 868	SMR 0.77, 95% CI 0.31–1.50
Bouchardy et al. 2002 [78]	Switzerland	Case–control	Incidence	1980–1993	129	9, 126	OR 1.20, 95% CI 1.00–1.50

^a^cohort size represents the total sample size in only cohort studies, and the total number of cases is only applicable to case–control studies

^b^HR – hazard ratio, SIR – standardized incidence ratio, SMR – standardized mortality/morbidity ratio, RR – relative risk, OR – odds ratio

**Table 3:** Characteristics of included studies on both firefighting and police work and prostate cancer risk (*N* = 7)

Author/Year	Location of Study	Study Design	Incidence or Mortality	Follow-up Period	Number of Cases/Deaths	Cohort Size/Total Number of Cases^a^	Prostate Cancer Risk Estimates for Ever versus Never Employment^b^
Sritharan et al, 2017b*	Canada	Cohort (linkage)	Incidence	1991–2011	165 firefighters; 325 police	1,100,000 1,100,000	HR 1.17, 95% CI 1.01–1.36; HR 1.22, 95% CI 1.09–1.36
Zeegers et al. 2004 [11]	Netherlands	Cohort (linkage)	Incidence	1986–1993	709 firefighters; 693 police	58, 279 58, 279	RR 0.59, 95% CI 0.05–6.33; RR 1.62, 95% CI 0.62–4.27
Demers et al. 1994 [28]	USA	Cohort	Incidence	1974–1989	66 firefighters; 28 police	2, 447 1, 878	SIR 1.40, 95% CI 1.10–1.70; IDR 1.10, 95% CI 0.70–1.80
Demers et al. 1992 [29]	USA	Cohort	Mortality	1945–1989	30 firefighters; 11 police	4, 546 3, 676	SMR 1.34, 95% CI 0.90–1.91; SMR 1.02, 95% CI 0.51–1.82
Sritharan et al. 2017a [79]	Canada	Case–control	Incidence	1995–1998	38 firefighters; 35 police	1, 737 1, 737	OR 1.67, 95% CI 0.94–2.95; OR 1.15, 95% CI 0.66–1.99
Sritharan et al. 2016 [80]	Canada	Case–control	Incidence	1994–1997	53 firefighters; 12 police	760 760	OR 0.73, 95% CI 0.53–1.01; OR 0.82, 95% CI 0.41–1.63
Sauve et al. 2016 [12]	Canada	Case–control	Incidence	2005–2009	26 firefighters; 45 police	1, 937 1, 937	OR 1.72, 95% CI 0.88–3.37; OR 1.60, 95% CI 1.00–2.40

^a^cohort size represents the total sample size in only cohort studies, and the total number of cases is only applicable to case–control studies

^b^HR – hazard ratio, SIR – standardized incidence ratio, SMR – standardized mortality/morbidity ratio, RR – relative risk, IDR – incidence density ratio, OR – odds ratio

*manuscript submitted and currently being revised for publication

Of all the firefighter studies, 2 pairs of studies (Ma et al., 2005 & Ma et al., 2006; Demers et al., 1992 & Demers et al., 1994) [26–29] examined the same respective populations but reported on different prostate cancer outcomes (incidence and mortality). In the meta-analyses of prostate cancer incidence and mortality in firefighters, respective results from both pairs of studies were retained and used. Two studies [30, 31] published results for both prostate cancer incidence and mortality, and each estimate was used [31, 32]. For the police studies, [28, 29] reported on the same populations with different outcomes of incidence and mortality, and each estimate was used. Each incidence and mortality outcome was used only in their respective categories and not included together for any meta-risk estimates.

### Quality assessment

The overall quality assessment of all 31 included studies ranged from 5 to 19 points (Table 4). Scores were similar for firefighter, police, and firefighter and police studies across the different quality assessment categories. The mean score for reporting was 6 out of 9, based on clear and detailed reporting of aims/hypotheses, outcomes measures, participant information, confounder information, and loss to follow-up. Studies were generally found to be externally valid, and there was minimal bias. Studies of firefighters had higher scores for confounding factors than studies of police workers. Only one study reported a power calculation making it difficult to evaluate this category.

**Table 4:** Quality assessment of included firefighter and police studies

Quality Assessment Category	Maximum Attainable Score	Studies on firefighters (*n* = 19)		Studies on police workers (n = 5)		Studies on both firefighters and police workers (n = 7)		All studies (*n* = 31)
		Range	Mean	Range	Mean	Range	Mean	Mean
Reporting	9	4–9	6.0	1–8	5.4	4–8	6.1	5.9
External Validity	2	1–2	1.8	0–2	1.6	1–2	1.6	1.7
Internal Validity: Bias	4	3–4	3.8	3–4	3.8	4	4	3.8
Internal Validity: Confounding	4	2–4	3.2	1–4	2.8	3–4	3.6	3.2
Power	1	0	0	0	0	0–1	0.1	0.0
Total	20	10–19	14.8	5–18	13.6	12–18	15.4	14.6

### Firefighter and prostate cancer meta-analyses

**Fig. 2:**
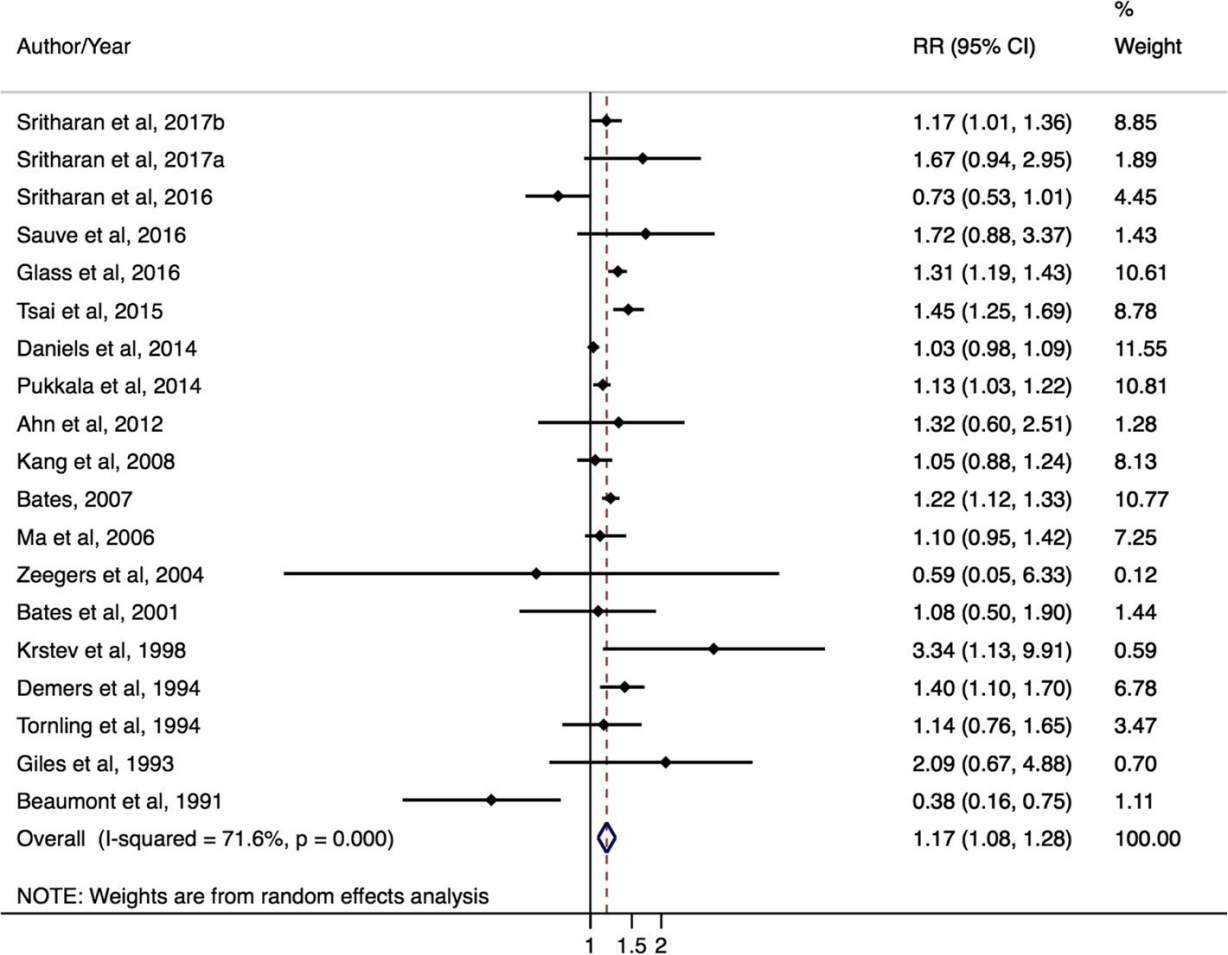
Forest plot and mRE of all included prostate cancer incidence studies on firefighters

**Fig. 3:**
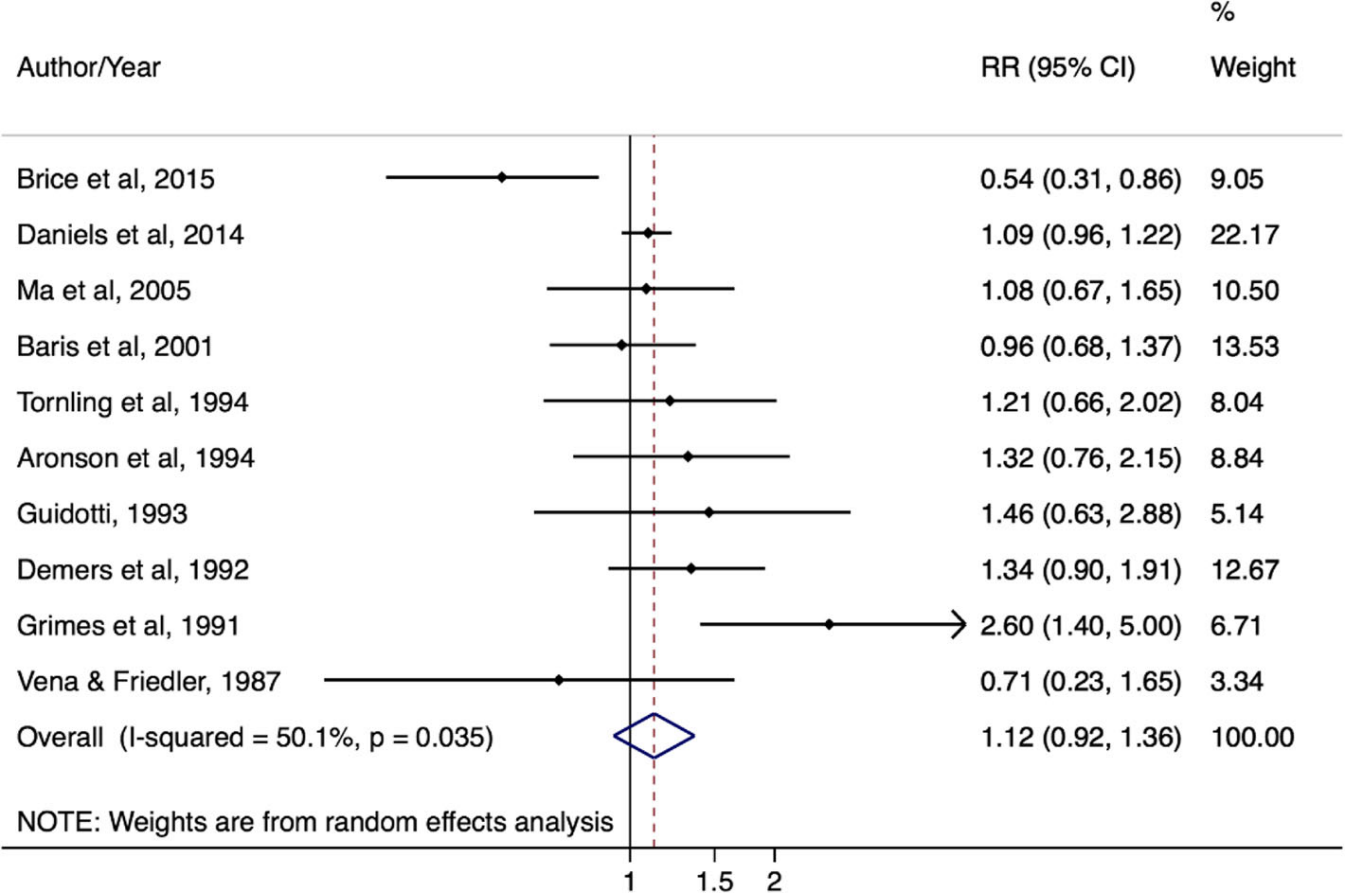
Forest plot and mRE of all included prostate cancer mortality studies on firefighters

There were significantly elevated prostate cancer risks for firefighting occupations for incidence outcomes, cohort studies, and administrative linkage-based studies. For incidence studies, the mRE was 1.17 (95% CI: 1.08–1.28; I^2^ = 72%, 95% CI: 55–82%, *p*-value <0.001; 19 studies) (Fig. 2); for mortality studies, it was 1.12 (95% CI: 0.92–1.36; I^2^ = 50%, 95% CI: 0–76%, p-value = 0.04; 10 studies) (Fig. 3). In cohort studies, the prostate cancer mRE was 1.14 (95% CI: 1.03–1.26; I^2^ = 67%, 95% CI: 46–80%, *p*-value <0.001; 18 studies) (Additional file 2: Figure S1). The meta-analysis of case–control studies resulted in an mRE of 1.27 (95% CI: 0.95–1.69; I^2^ = 78%, 95% CI: 53–90%, *p*-value <0.001; 6 studies) (Additional file 3: Figure S2). The mRE for census or administrative linkage-based studies was 1.19 (95% CI: 1.06–1.34; I^2^ = 61%, 95% CI: 0–85%, *p*-value = 0.04; 5 studies) (Additional file 4: Figure S3).

### Police work and prostate cancer meta-analyses

**Fig. 4:**
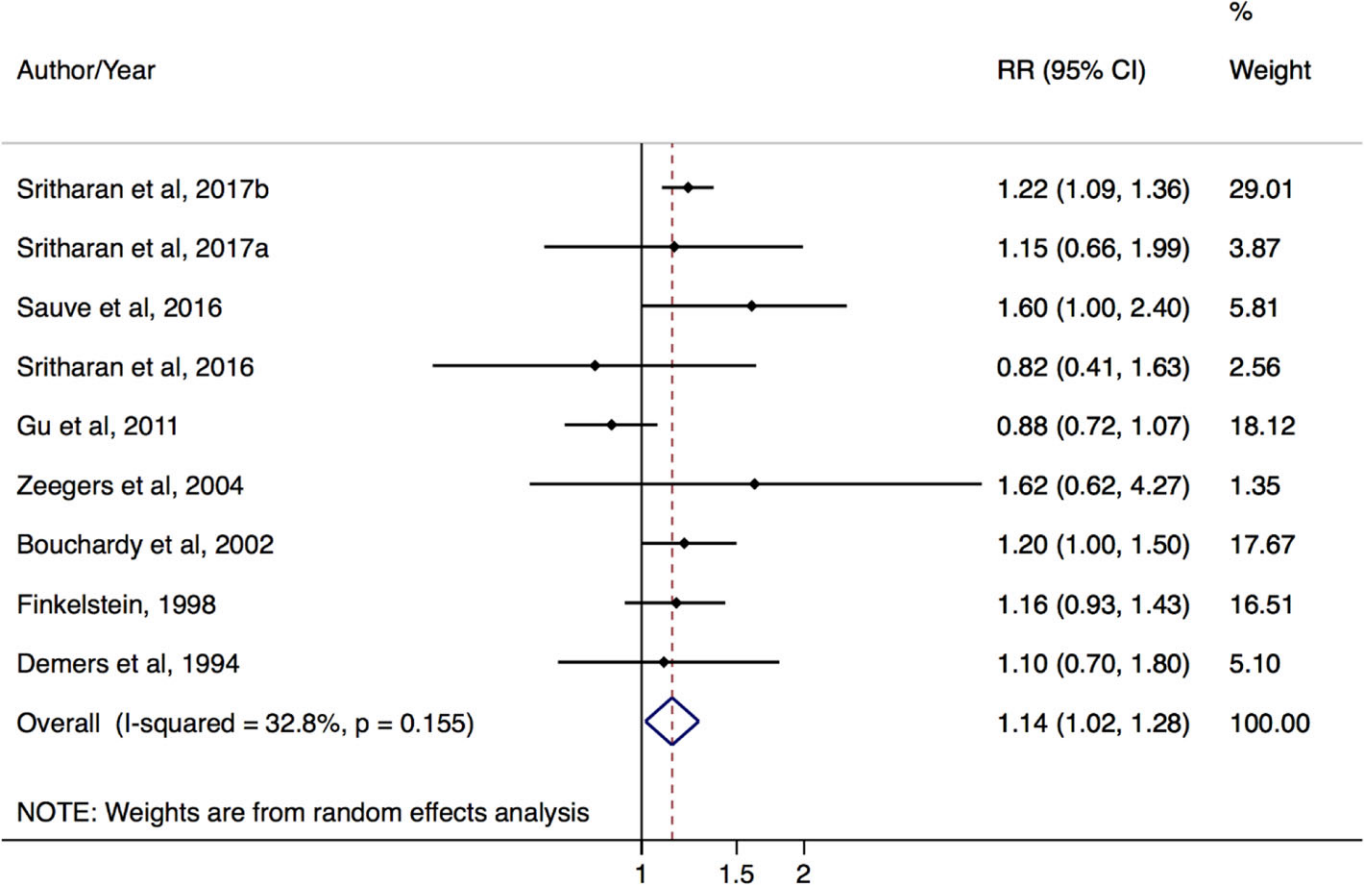
Forest plot and mRE of all included prostate cancer incidence studies on police workers

**Fig. 5:**
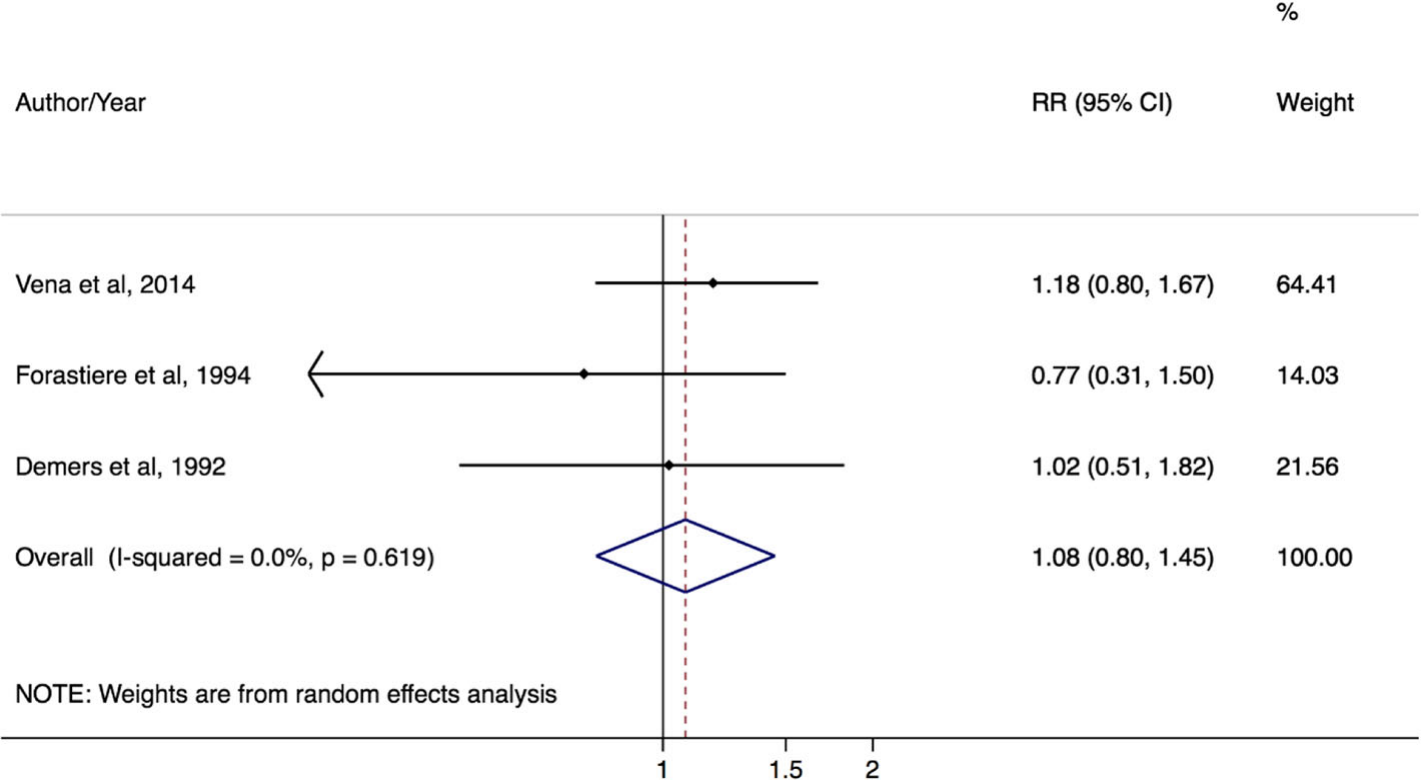
Forest plot and mRE of all included prostate cancer mortality studies on police workers

There were significantly elevated prostate cancer risks for police occupations by incidence outcomes and in case–control studies. The mRE for prostate cancer incidence studies was 1.14 (95% CI: 1.02–1.28; I^2^ = 33%, 95% CI: 0–74%, *p*-value = 0.16; 9 studies) (Fig. 4) while the mRE for prostate cancer mortality studies was 1.08 (95% CI: 0.80–1.45; I^2^ = 0%, 95% CI: 0%–90%, *p*-value = 0.62; 3 studies) (Fig. 5). The mRE for case–control studies was higher compared to the mRE for cohort studies (case–control studies: mRE = 1.22, 95% CI: 1.03–1.44; I^2^ = 0% (95% CI 0%–85%, p-value = 0.42; 4 studies) (Additional file 5: Figure S4) versus cohort studies: mRE = 1.10, 95% CI: 0.96–1.26; I^2^ = 37%, 95% CI: 0–79%, p-value = 0.15; 7 studies) (Additional file 6: Figure S5). There were no administrative linkage-based studies of police workers and prostate cancer risk.

### Between-study heterogeneity

There was high heterogeneity (72%) for the meta-analysis of all 19 firefighter incidence studies. As a sensitivity analysis, the Galbraith plot was used and one study [33], appeared outside of the 95% confidence region. Removal of this study resulted in a minimal change in heterogeneity (72 versus 69%, respectively). For the meta-analysis of the 10 mortality studies, there was moderate heterogeneity (50%). High heterogeneity was observed for the six case–control studies (78%), 18 cohort studies (67%) and the five administrative linkage-based studies (61%). When plotting these subgroups using the Galbraith plot, no studies appeared outside of the 95% confidence region.

For police studies, heterogeneity ranged from none to moderate. Moderate heterogeneity was observed for the nine incidence studies (33%) and seven cohort studies (37%), but no heterogeneity (0%, 95% CI: 0–90%) was observed for the mortality (three studies) and case–control (four studies) subgroups. I^2^ values of 0% are biased and imprecise, likely because of the small number of studies in these subgroups (*n* < 5) [34]. Using the Galbraith plot, none of the police studies appeared outside of the 95% confidence region.

### Publication bias

There was no evidence of publication bias according to Begg’s test (*p* = 0.86) and Egger’s test (*p* = 0.11) for the meta-analysis of all 19 firefighter incidence studies. No publication bias was evident for the 9 police incidence studies (Begg’s test: *p* = 0.60, Egger’s test: *p* = 0.68). There were also no statistically significant findings for publication bias for mortality studies, case–control, cohort, and administrative linkage-based studies.

## Discussion

In this meta-analysis of 31 epidemiological studies of protective services workers, nearly identical and small statistically significant excess risks of prostate cancer were found for ever working in firefighting and police work. Statistically significant and borderline prostate cancer mREs were found for firefighters in separate evaluations of incidence studies, cohort studies, and administrative linkage studies, as well as in each meta-analysis of police worker incidence studies and case–control studies. Most studies were of average quality, with opportunities for improvement in reporting and study power assessment. As expected, case–control studies compared to cohort studies generally had more information on variables that can act as potential confounders of the firefighter/police work and prostate cancer associations. All case–control studies reported prostate cancer risk estimates that were adjusted for age; most were also adjusted for ethnicity. Fewer case–control studies adjusted risk estimates for family history of prostate cancer and potentially confounding variables such as socioeconomic status, physical activity, height, obesity, active smoking, and alcohol consumption. Overall, findings from this meta-analysis support positive associations found between prostate cancer risk and firefighting in the epidemiological literature, and indicate a potential relationship with police work as well.

There are a few hypotheses that may explain why employment in protective services occupations could be associated with increased prostate cancer risk. Firefighting and police jobs are inherently dangerous occupations that involve stressful, and, at times, life-threatening, situations with exposure to multiple hazards [14–16]. Psychological stressors can influence biological processes and lead to decreased immune function, increased pro-inflammatory cytokine secretion, and cancer progression [15]. Shift work, which is common in protective services work, was significantly associated with increased prostate cancer risk in a recent meta-analysis of eight case–control and cohort studies [35]. Firefighters are also exposed to toxins released by fire and smoke including benzene, 1,3-butadiene, formaldehyde and at times can be exposed to other compounds such as radiation, diesel exhaust, asbestos, metals (arsenic and cadmium), and PAHs [14, 16, 27]. The chemical reactions during combustion and the age and type of building or material on fire can contribute to exposure to these compounds [16]. Police work involves fewer chemical exposures compared to firefighting, although exposure to ionizing radiation from radar devices is a concern for overall cancer risk [11, 12]. Firefighters and police workers may also be exposed to air pollution on the job, as ambient concentrations of ultrafine particles and NO_2_ have been previously linked to prostate cancer risk [36, 37]. Of the described chemical exposures, only x and gamma radiation, arsenic compounds, and cadmium compounds have been linked to prostate cancer by IARC based on limited evidence in non-occupational settings. However, IARC has classified benzene, ionizing radiation, diesel exhaust, asbestos, arsenic compounds, cadmium compounds, and air pollution as all Group 1 carcinogens, based on evidence for other cancer sites [38]. There is a need to further examine these chemical exposures in both firefighting and police work to understand if these exposures are involved in prostate cancer risk.

Evaluating potential associations between shift work and prostate cancer is an active area of ongoing research [39–41]. Shift work can disrupt the body’s endogenous circadian rhythm (sleep-wake cycle) and contribute to increased susceptibility to acute and chronic diseases. However, the biological mechanisms that may be involved in prostate and other cancers have not been established [18, 42]. One hypothesis is that night shift work can lead to decreased melatonin, which can then lead to continuous testosterone production, influencing the growth and differentiation of prostate cancer cells [16]. In addition, decreased sunlight exposure in night shift workers reduces the production of vitamin D, thereby compromising the effects of vitamin D on suppressing the production of prostate cancer cells [16].

Psychological stress also has been linked to cancer progression, but there is limited evidence for how this impacts cancer promotion [43]. Firefighting and police work involve constant stressors that can potentially affect cancer progression, particularly prolonged stress over years of employment in these jobs [44]. A recent study on stress at work and cancer outcomes found that the highest prevalence of stress at work was reported among firemen when compared to other types of occupations [45].

Another factor that may influence our meta-analysis results is prostate cancer screening. Although prostate specific antigen (PSA) testing varies across different countries and within countries, it is believed that protective services workers have frequent and better access to health resources compared to other workers, including access to cancer screening [32]. In North America, for example, firefighters are provided with health information and recommendations on what to consider when completing a health examination with their primary physician, including recommendations for prostate cancer screening [46]. However, it is up to each fire department to disseminate this information and ultimately up to each firefighter to request screening from their primary physician. In this meta-analysis we found slightly lower mortality mREs compared to incidence mREs for firefighters and police officers. As increased screening of prostate cancer leads to the identification of more early stage cases (increased incidence), this may be indicative of a screening effect. However, the mREs for both incidence and mortality were so similar that it was difficult to attribute these differences to screening. Also, prostate cancer screening may not be of high importance in firefighting compared to other cancers (ex. brain, bladder, and colon) and health conditions that have been consistently associated with firefighting. We evaluated study estimates based on different follow-up periods defined as pre-PSA period (prior to 1990 before the PSA test was introduced), during the introduction of PSA testing (early 1990s), and after the introduction of PSA testing (late 1990s and onwards). Although we included studies from different nations, most of the studies were North American so we loosely defined the time periods based on North America. We identified a number of pre-PSA period firefighter studies and observed a meta-risk estimate of 1.26 (95% CI 0.96–1.67) for these studies. For firefighter studies that had follow-up periods during and after the introduction of PSA testing, we observed a meta-risk estimate of 1.13 (95% CI 1.02–1.25). It was challenging to define firefighter study follow-up periods as post PSA testing (late 1990s onwards) since most of these studies had follow-up periods that overlapped the early 1990s when PSA testing began. We identified only a few firefighter studies that had later follow-up periods (late 1990s and early 2000s) and observed a meta-risk estimate of 1.58 (95% CI 1.09–2.29) for these studies. Overall, we observed an elevated risk for firefighter studies that were conducted before the introduction of PSA testing, and a statistically significant elevated risk for firefighter studies that took place during and after the introduction of PSA testing. These findings may be representative of the increased screening that took place over this time period. We attempted to evaluate police studies as well but were limited as almost all included police studies had follow-up periods overlapping periods with and without PSA testing.

Our findings of a slight excess risk of prostate cancer in firefighting and police services should be cautiously interpreted. As expected, there was considerable heterogeneity between studies, particularly in subgroup meta-analyses of police workers and prostate cancer risk that involved small numbers of studies. This makes it challenging to interpret mRE values with precision [34]. Heterogeneity was likely due to differences in study design and populations studied, follow-up years, occupational exposure assessment and job coding, and adjustment of relative risk values for known or potential covariates. Specifically, there were differences in how the study populations were defined, in terms of paid or unpaid work, full time vs. part time, and eligible employment duration. Some heterogeneity may also be attributed to different follow-up periods in each study, especially those overlapping the pre and post PSA era. The variation in age distribution across included studies could also contribute to heterogeneity based on differences in how studies stratified by age. Some studies had relatively younger populations than other studies and we observed a similar elevated meta-risk estimate for these younger population studies as we did for the overall estimates Publication bias was also considered, but was not recognized as a significant factor as a majority of the included studies were cohort designs. The cohort studies generally looked at multiple cancer sites as outcomes, so it is unlikely that publication bias would have been of concern based on solely prostate cancer results.

A major strength of this meta-analysis is that it was the first to assess prostate cancer risk in both firefighting and police work, replete with subgroup analyses and assessments of study quality, heterogeneity, and publication bias. This meta-analysis captured all previously and newly published studies since the IARC evaluation of firefighting in 2007, and also quantitatively evaluated prostate cancer risk in police studies which had not been done before. Firefighting and police work should be priority areas for investigation because these occupations frequently involve exposure to multiple chemical, biological, physical, and psycho-social hazards. Exposure to some hazards may be associated with increased risk of prostate cancer, although the strength and consistency of associations varies across studies and there are substantial research gaps. Altogether, this research can be used to help identify opportunities for further research on occupation and prostate cancer risk.

Other occupations of interest with respect to prostate cancer risk are military workers. While we initially sought to include military studies in this meta-analysis, they were ultimately not included because these studies were primarily based on specific historical events (ex. Gulf war) or internal comparisons between military groups [47–62]. This made it difficult to compare findings to other studies that did not focus on single events or that compared workers to the general population. Future assessments can separately consider military studies.

## Conclusions

Overall, the slight excess risks of prostate cancer in firefighting and police services found in this meta-analysis of 31 studies were generally robust to subgroup analyses by outcome (incidence and mortality) and study design. Our findings are important as they show the importance of prostate cancer incidence and mortality among protective services workers, and as this is the first meta-analysis to include both firefighting and police work and prostate cancer risk. The observed findings suggest that screening may not entirely explain our findings, but further investigation into actual screening rates and screening behaviours in firefighting and police work is warranted. Also, further investigations should be designed to assess specific exposures such as benzene, radiation, diesel exhaust, arsenic and cadmium compounds, PAHs, asbestos, and air pollution which are involved in firefighting. Little evidence on how they may relate to prostate cancer risk has been accrued. There is also a need for future studies to examine prostate cancer risk in police work given the small number of police workers published to date. By addressing these important issues in future studies, there will be better understanding on prostate cancer risk in firefighting and police work.

## Additional files

Additional file 1: Table S1. Covariates adjusted for in firefighter and police case–control studies (DOCX 12 kb)

Additional file 2: Figure S1. Forest plot and mRE of all included cohort studies on firefighters. (DOCX 285 kb)

Additional file 3: Figure S2. Forest plot and mRE of all included case–control studies on firefighters. (DOCX 137 kb)

Additional file 4: Figure S3. Forest plot and mRE of all included administrative linkage-based studies on firefighters. (DOCX 153 kb)

Additional file 5: Figure S4. Forest plot and mRE of all included cohort studies on police workers. (DOCX 175 kb)

Additional file 6: Figure S5. Forest plot and mRE of all included case–control studies on police workers. (DOCX 117 kb)

## Abbreviations

CI: Confidence Interval
HR: Hazard Ratio
IARC: International Agency for Research on Cancer
mRE: Meta-risk Estimate
OR: Odds Ratio
PSA: Prostate Specific Antigen
RR: Relative Risk
SIR: Standardized Incidence Ratio
SMR: Standardized Mortality Ratio
