# Supplementary material for: Prostate cancer in firefighting and police work: a systematic review and meta-analysis of epidemiologic studies
Source: Environ Health. 2017 Nov 17;16:124. doi: 10.1186/s12940-017-0336-z (PMC5693511; doi:10.1186/s12940-017-0336-z)
Supplement: Supplementary file 1 — Covariates adjusted for in firefighter and police case–control studies (DOCX 12 kb) [file 12940_2017_336_MOESM1_ESM.docx]

**Additional Files (Supplementary)**

**Additional File 1. Table S1. Covariates adjusted for in firefighter and police case-control studies**

| **Author (Year)** | *Age* | *Ethnicity* | *Family history of prostate cancer* | *Socioeconomic  status proxy  (income or education)* | *Physical activity* | *Obesity  (BMI)* | *Height* | *Smoking* | *Alcohol Intake* |
| --- | --- | --- | --- | --- | --- | --- | --- | --- | --- |
| Sritharan et al (2017a) | Y | Y | Y | Y | Y | Y | N | Y | N |
| Sauve et al (2016) | Y | Y | Y | Y | Y | Y | N | N | Y |
| Sritharan et al (2016) | Y | Y | Y | N | N | N | N | N | N |
| Tsai et al (2015) | Y | Y | N | N | N | N | N | N | N |
| Kang et al (2008) | Y | N | N | N | N | N | N | Y | N |
| Bouchardy et al (2002) | Y | N | N | Y | N | N | N | N | N |
| Krstev et al (1998) | Y | Y | N | N | N | N | N | N | N |

*studies shown are only case-control designs
