# Supplementary figures and images for: Prostate cancer in firefighting and police work: a systematic review and meta-analysis of epidemiologic studies
Source: Environ Health. 2017 Nov 17;16:124. doi: 10.1186/s12940-017-0336-z (PMC5693511; doi:10.1186/s12940-017-0336-z)

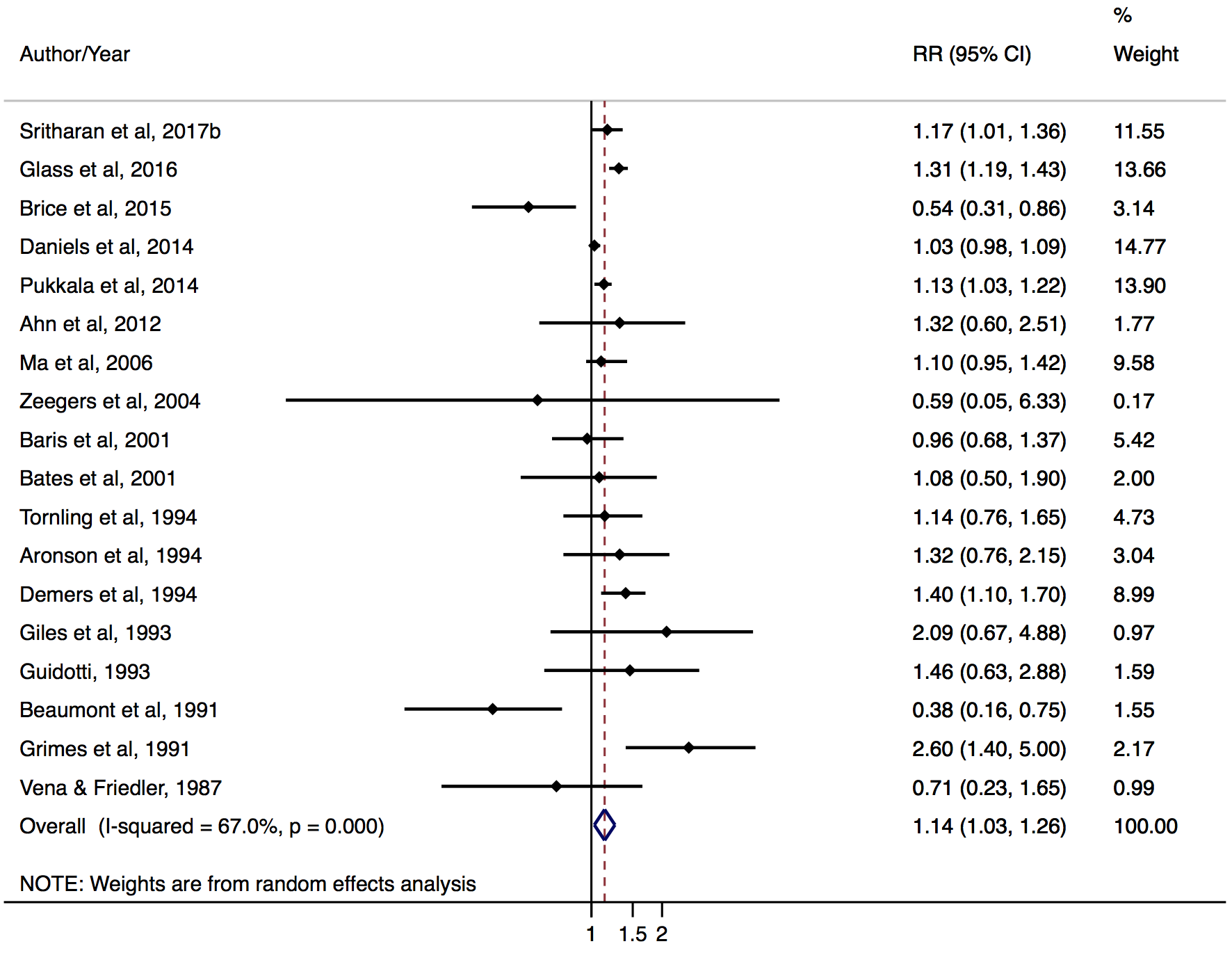

Supplement: Supplementary file 2 — Forest plot and mRE of all included cohort studies on firefighters. (DOCX 285 kb) [file 12940_2017_336_MOESM2_ESM.docx]

**
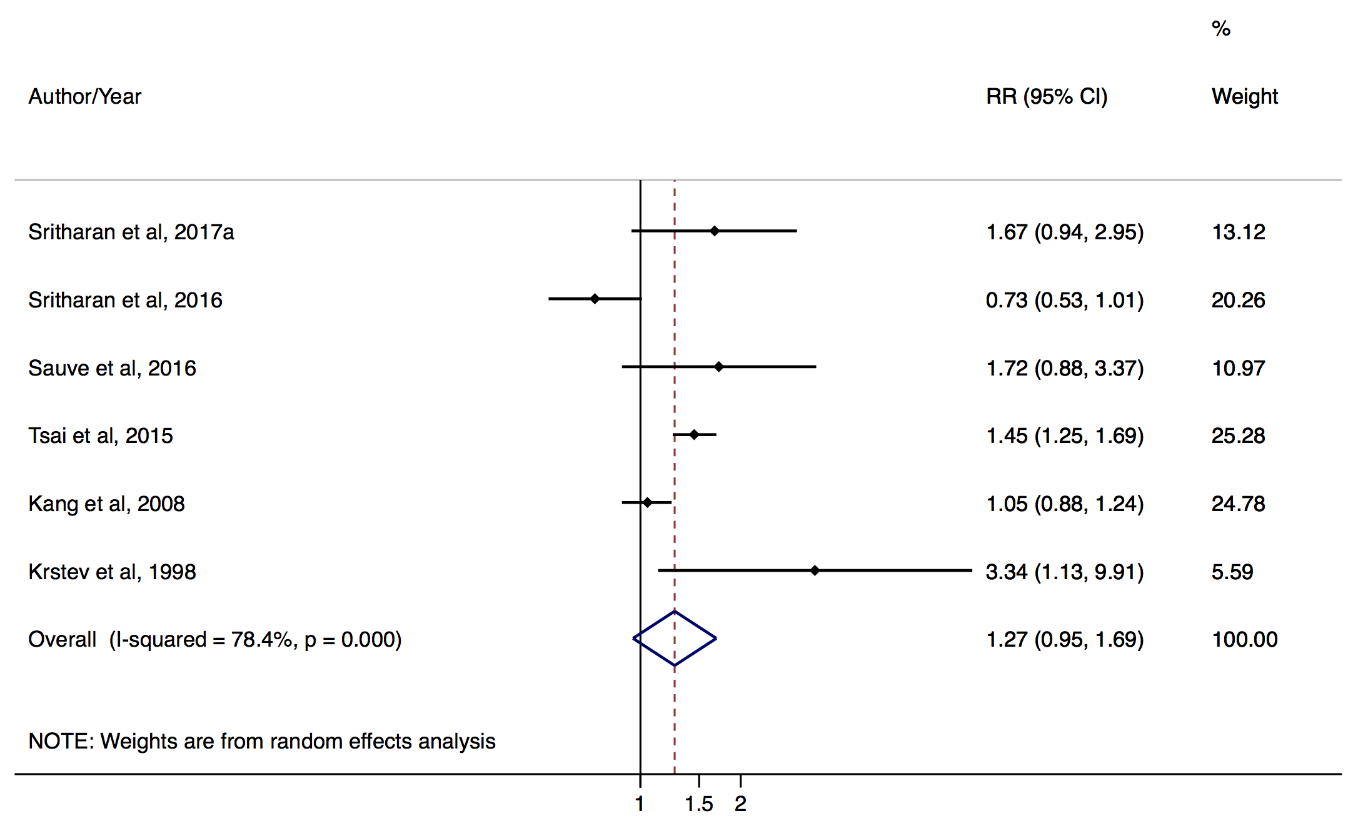
**

Supplement: Supplementary file 3 — Forest plot and mRE of all included case–control studies on firefighters. (DOCX 137 kb) [file 12940_2017_336_MOESM3_ESM.docx]

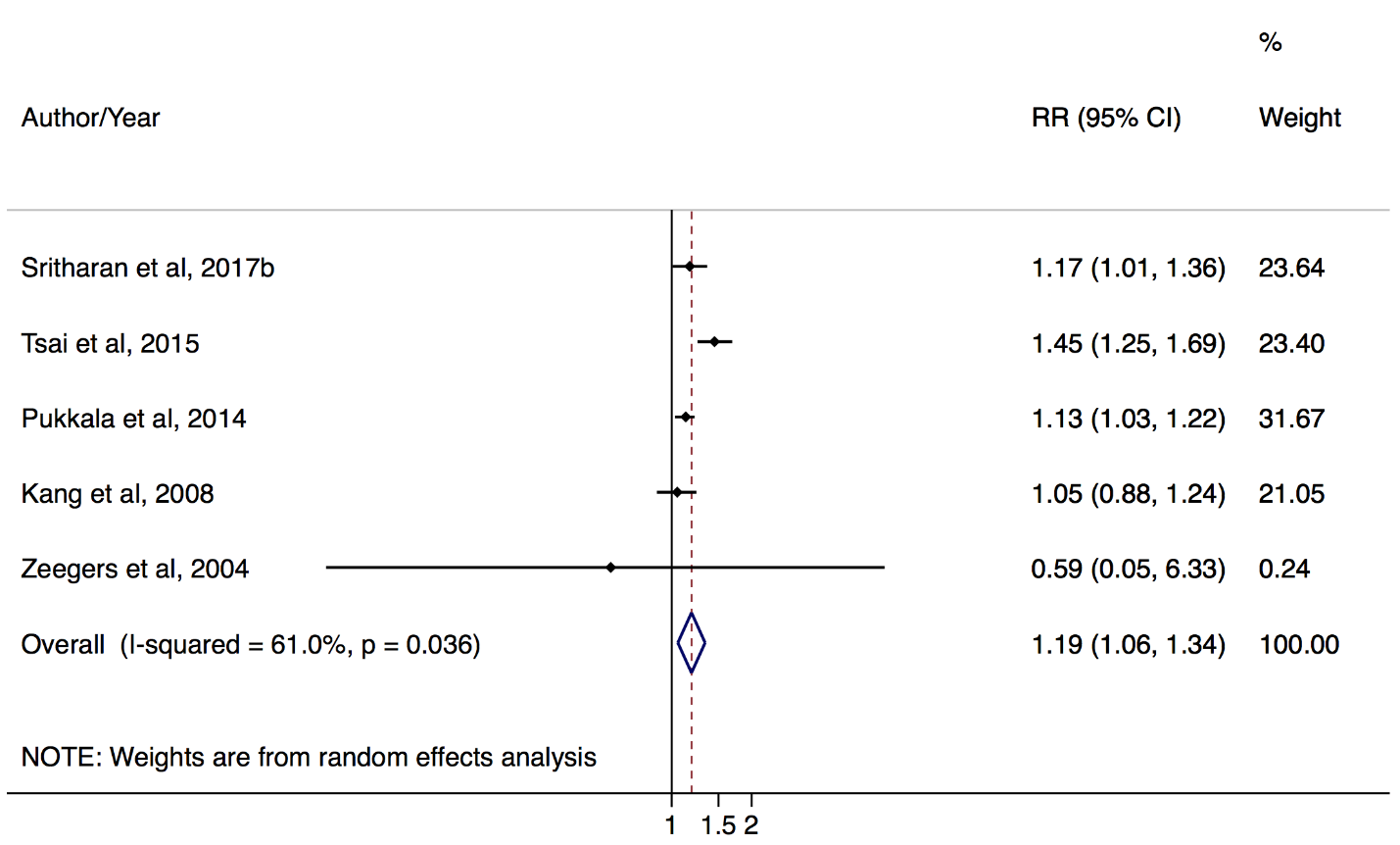

Supplement: Supplementary file 4 — Forest plot and mRE of all included administrative linkage-based studies on firefighters. (DOCX 153 kb) [file 12940_2017_336_MOESM4_ESM.docx]

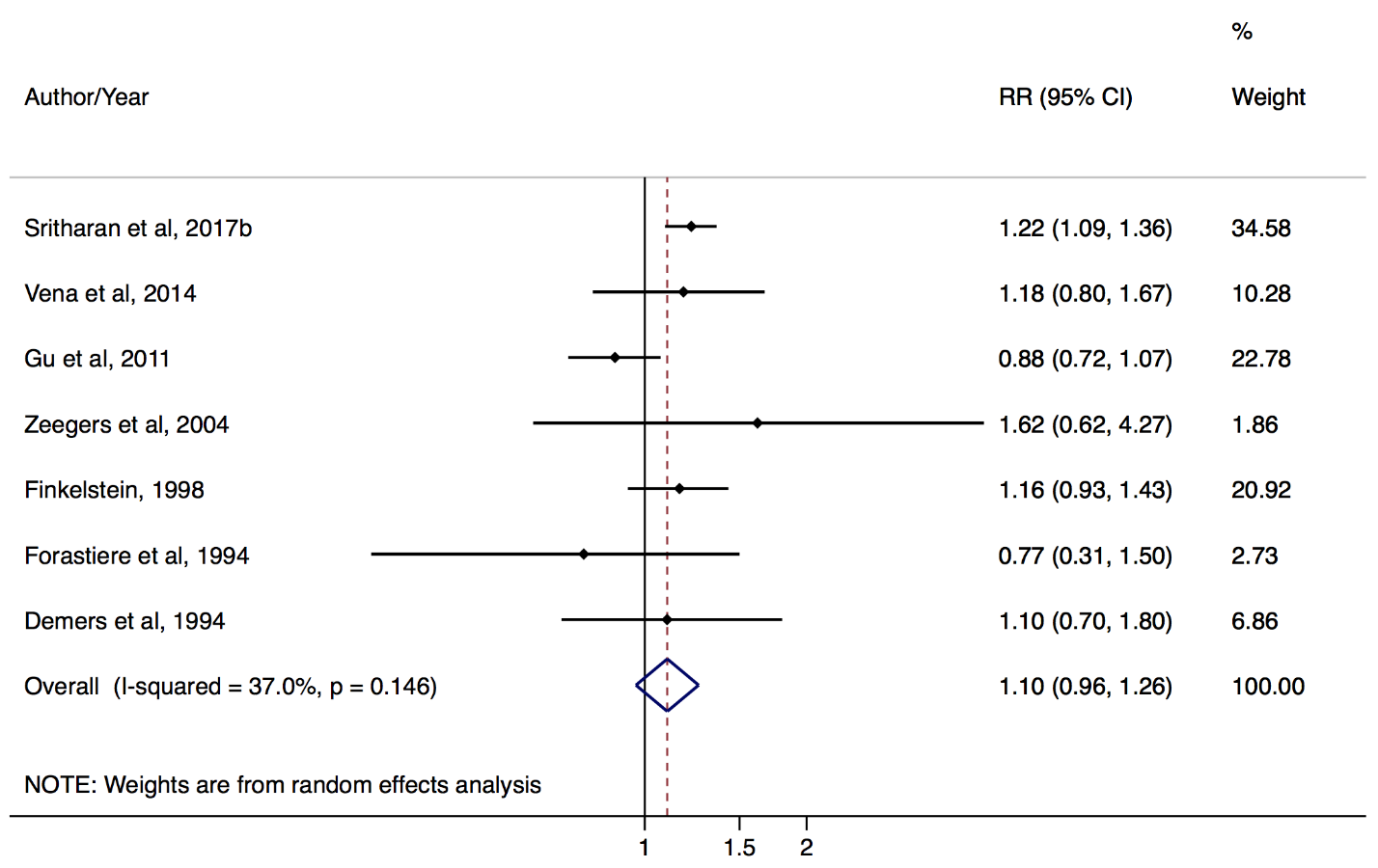

Supplement: Supplementary file 5 — Forest plot and mRE of all included cohort studies on police workers. (DOCX 175 kb) [file 12940_2017_336_MOESM5_ESM.docx]

**
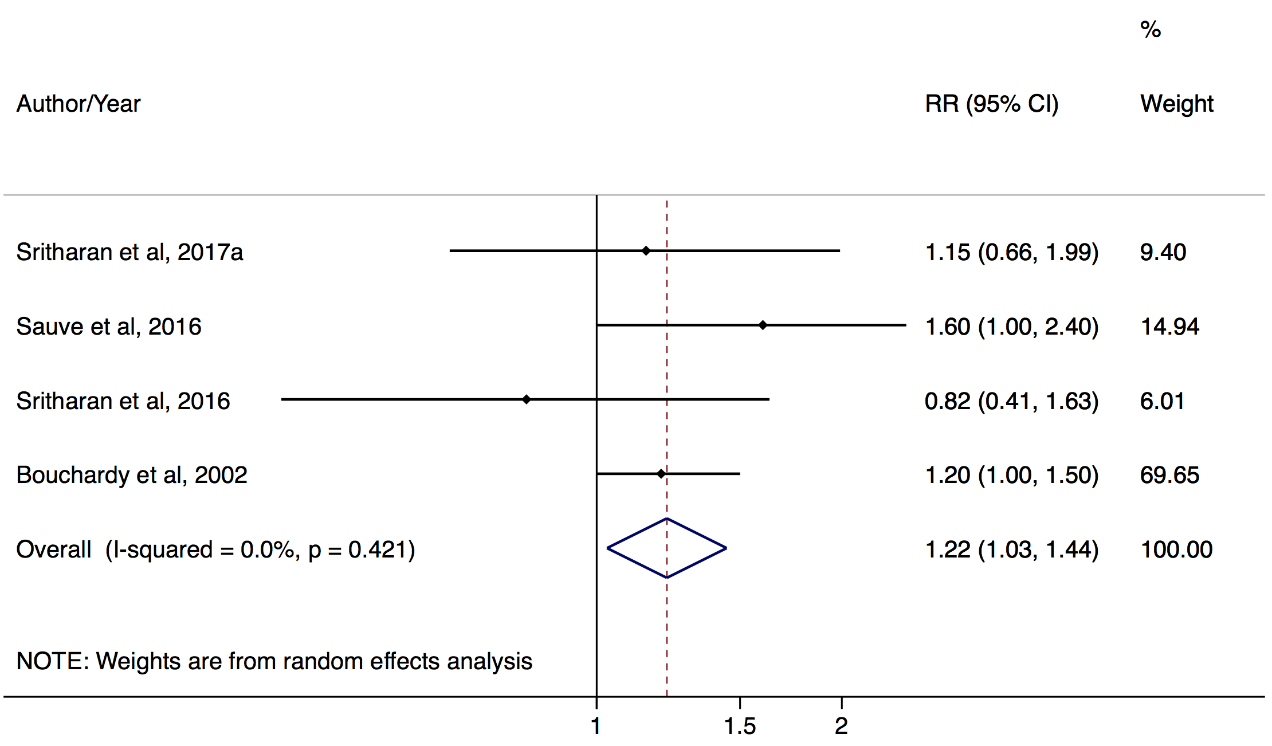
**

Supplement: Supplementary file 6 — Forest plot and mRE of all included case–control studies on police workers. (DOCX 117 kb) [file 12940_2017_336_MOESM6_ESM.docx]
